# Middle Triassic Limestones as a Source of Trace Elements and REY

**DOI:** 10.3390/ma17153668

**Published:** 2024-07-25

**Authors:** Katarzyna Stanienda-Pilecki, Katarzyna Nowińska, Andrzej Nowrot, Janusz Szewczenko

**Affiliations:** 1 Faculty of Mining, Safety Engineering and Industrial Automation, Silesian University of Technology, ul. Akademicka 2, 44-100 Gliwice, Poland; katarzyna.stanienda-pilecki@polsl.pl (K.S.-P.); andrzej.nowrot@polsl.pl (A.N.); 2 Faculty of Biomedical Engineering, Silesian University of Technology, ul. Roosevelta 40, 41-800 Zabrze, Poland; janusz.szewczenko@polsl.pl

**Keywords:** Triassic limestones, trace elements, REY

## Abstract

The results of the content determination of the selected trace elements Ti, Sr, Ba, Zn, Pb, Cu, S, P, Cr, Cd, Ni, Zr, Mo, Rb, Sc, Y, and REEs were presented in this article. Studies were carried out to define the purity of limestones. The elements were measured in the carbonate minerals of Middle Triassic limestones of the Opole Silesia in Poland, using ICP-MS and X-ray fluorescence (XRF). Moreover, the contents of Sc and REY were also determined by electron microprobe analysis. These methods are characterized by high accuracy and precision of the measurement. The research results show that the contents of trace elements vary from below 1 ppm up to some hundreds ppm. The highest content was measured for strontium and the lowest for barium, elements characteristic of aragonite. Their occurrence indicates the presence of aragonite in the primary carbonate material. Some trace elements form substitutions in clay or carbonate minerals. Zn, Pb, Cu, Mo, and Ni may be associated with sulfides, and Ti and Cr may be associated with oxides. Sc and REY usually substitute Ca ions in calcite. The contents of measured Ce, Nd, Sm, Dy, Er, and Y vary from below 1 ppm up to 6 ppm, and Sc from 0 ppm to 10 ppm.

## 1. Introduction

Limestones are sedimentary rocks used in various branches of industry, such as the lime industry and the fertilizer industry. It is also used in building construction, road construction, cement production, animal feed additives, sorbent for desulfurization of flue gases, and others. Limestones are usually built of low-magnesium calcite minerals (CaCO_3_). In some limestones or other carbonate rocks, other carbonate phases occur, such as high magnesium calcite (high-Mg calcite), proto-dolomite, ordered dolomite, and huntite [1,2,3,4,5,6,7,8,9].

The results of the previous research have shown that very often in limestones, some non-carbonate impurities are present, including different trace elements, such as Sc and REY (rare earth elements—REEs and yttrium together are referred to as REY) [4,6,10,11,12,13,14,15,16,17,18]. Therefore, the study of Triassic carbonate rocks situated in the area of Opole Silesia in the SW part of Poland was undertaken to determine whether the trace elements, Sc and REY, are present in these rocks and in what quantities.

Trace elements usually appear in small amounts. The most common trace elements present in limestones are Ti, Sr, Ba, Zn, Pb, Cu, S, P, Cr, Cd, Mo, Ni, Rb, Zr, Nb, and Hf. Sometimes, Sc and REY (REEs and Y) may also occur [6,9,16,18]. Very often, Sr and Ba are present in calcite. However, these elements occur in aragonite [10,11,19,20,21,22]. Sr and Ba substitute Ca in this carbonate phase. Because aragonite is transformed, such as high-Mg calcite into low-Mg calcite, during diagenesis, these elements occur in calcite. The presence of strontium and barium, however, indicates that the original carbonate material included aragonite [10,11,19,20,21,22]. The other trace elements may occur as substitutions in clay or carbonate minerals. Zn, Pb, Cu, Mo, and Ni can also be bound in sulfides, and Ti, Cr, and Mn in oxides.

Sc and REY—rare earth elements (REEs) and Y (yttrium) elements—are valuable indicators of provenance and alteration for minerals and rocks. They provide invaluable information about the environment and processes that influence mineral formation and growth as well as the circumstances characterizing subsequent alteration [13,18,23,24]. Ca-bearing minerals are commonly enriched in REEs, Y, and Sc relative to other phases, reflecting the ease with which REEs, Sc, and Y substitute for Ca [13,23,24]. The REEs, Sc, and Y usually occur as trivalent ions and show similar chemical behaviors owing to their electron configurations. Carbonate Sc, Y, and REE values are problematic, particularly according to the diagenetic change.

The subject of research was Triassic limestones (Lower Muschelkalk) from the area of Opole Silesia which is situated in the South-West part of Poland [6,9]. The full profile of Middle Triassic—Muschelkalk was found there. The Lower Muschelkalk profile contains Gogolin Beds (the bottom of the profile—initial sea transgressive stage), Górażdże Beds (advanced sea transgression stage), Dziewkowice (Terebratula) Beds (sea transgression peak stage) and Karchowice Beds (the upper formation of profile—sea regression stage) [25].

The study results of Ti, Sr, Ba, Zn, Pb, Cu, S, P, Cr, Cd, Ni, Zr, Mo, Rb, Nb, and Sc and REY content, measured in the carbonates of the Muschelkalk (Middle Triassic) limestone samples taken from deposits situated in the area of the Opole Silesia in Poland, were presented in this article. The research was carried out to find out whether the content of the examined elements is similar or different in the limestones of each formation. It will also be possible to say whether the content of these elements is related to the stages of marine transgressions or regression. The presence of some trace elements may also be associated with the mineralization of rocks during hydrothermal processes, like in the areas of Upper Silesia and Kraków [26,27]. The purpose of the research was to determine the content of trace elements, Sc and REY, in Triassic limestones. The results of the research allowed us to establish the origin of trace elements, Sc and REY, in limestones in terms of the formation of these rocks and the processes of diagenesis and other processes that influenced their final geochemical composition. The results of the research will provide data on the possible harmful impact on the environment of trace elements present in carbonate rocks and the products of the processing of these rocks. Moreover, based on the measured Sc and REY content, it will be possible to determine whether it would be possible to recover these elements from limestone. This would, of course, have to be economically justified.

## 2. Materials and Methods

### 2.1. Materials

**Figure 1:**
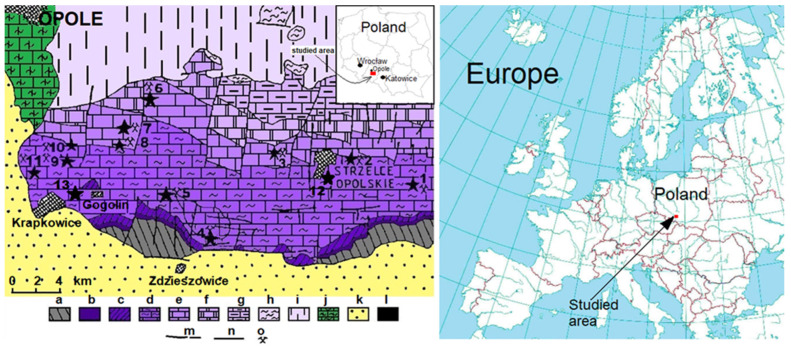
Geological map of the central part of Opole Silesia, according to Niedźwiedzki [28]; a—greywackes of Lower Carboniferous; b—sandstones and mudstones of Middle Buntsandstein; c—limestones, dolomites, and marls of Upper Buntsandstein (Roethian); d—limestones and marls of Gogolin Beds; e—limestones of Górażdże Beds, Terebratula Beds, and Karchowice Beds; f—dolomites of Jemielnice Beds; g—limestones and dolomites of Rybna Beds and Boruszowice Beds; h—claystones, mudstones, and sandstones of Keuper; i—claystones of Rhaetian; j—sandstones, marls, and limestones of Upper Cretaceous; k—sandstones, clays, and gravels of Neogene; l—Tertiary basalts; m—faults; n—stratigraphic boundaries; o—important quarries: 1–11—quarries and outcrops: 1—Błotnica Strzelecka; 2—Dziewkowice; 3—Szymiszów; 4—Saint Anne Mountain and Wysoka; 5—Ligota Dolna and Kamienna; 6—Tarnów Opolski; 7—Kamień Śląski; 8—Górażdże and Kamionek; 9—Malnia; 10—Chorula; 11—Rogów Opolski; 12—Strzelce Opolskie; 13—Gogolin, 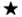
—areas of sampling.

The analyzed area is located in Opole Silesia, which is between Lower Silesia (the main city is Wrocław) and Upper Silesia (the main city is the Katowice urban area). The studied area is located exactly southeast of Opole (the city and capital of Opole Province) (Figure 1).

**Figure 2:**
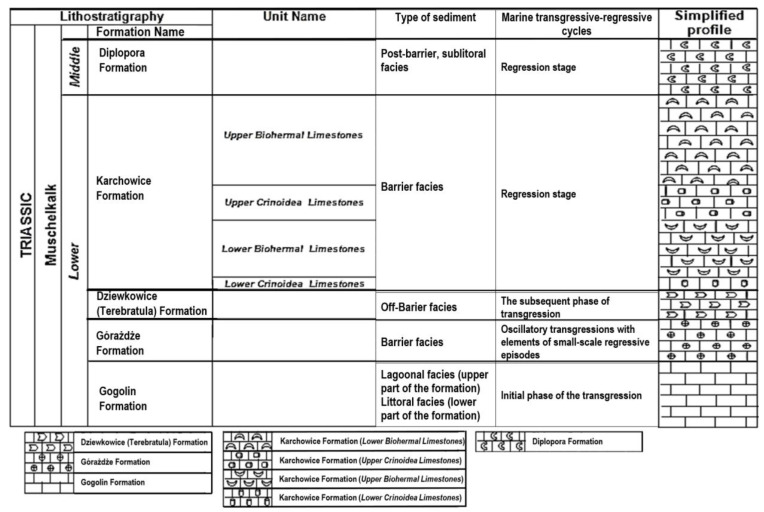
Simplified geological profile of the Middle Triassic (Muschelkalk) carbonate sediments in the central part of Opole Silesia, where Triassic (from ~251.9 to ~204.1 mln years ago); Lower (from 247.2 to ~242 mln years ago); and Middle (from ~242 to ~237 mln years ago) [author of the figure Stanienda-Pilecki K].

The samples for laboratory tests were collected in Gogolin Quarry, Gogolin, Poland (samples G1, G6; Figure 1(13)), Ligota Dolna Quarry, Ligota Dolna, Poland (sample LD11; Figure 1(5)), Wysoka Quarry, Niegowonice, Poland (samples W1, W5; Figure 1(4)), Szymiszów Quarry, Szymiszów, Poland (sample S2; Figure 1(3)), Strzelce Opolskie Quarry, Strzelce Opolskie, Poland (samples SO1, SO14, SO17, SO20; Figure 1(12)) and the area of Saint Anne Mountain, Poland (sample SA5, SA12, CZ1, CZ2, CZ3, CZ4 and CZ5; Figure 1(4)). In total, 17 samples were studied: 3 samples from the Gogolin Formation (G1, G6, LD11), 3 samples from the Górażdże Formation (W1, W5, SA5), 3 samples from the Terebratula (Dziewkowice) Formation (SA12, S2, SO1), 3 samples from the Karchowice Formation (SO14, SO17, SO20), and 5 samples from the contact zone between Triassic limestones and Paleogene-Neogene basalts from the area of Saint Anne Mountain (CZ1, CZ2, CZ3, CZ4 and CZ5) (Figure 1 and Figure 2, Table 1) [6,9,28].

The Gogolin Formation includes two types of sediments: littoral facies (the lower part of the formation) and lagoonal facies (the upper part of the formation) (Figure 2). They are sediments of the initial phase of the sea transgression [25,29]. The sediments of the Gogolin Formation were formed in shallow marine well-aerated environments, strongly affected by storm events [25]. The layers of the lower part of the Gogolin Formation are deposited in the eulittoral and sublittoral zones. The layers of the upper part of the formation are off-barrier deposits of the sublittoral zone [29]. They are usually medium-bedded, organodetrital. Some of the Gogolin limestones are marly and others are sandy [29]. The lower part of this formation—littoral facies—is built of sediments formed in a shallow zone. During a short period of time, hypersaline conditions were developed here and evaporation occurred. It led to subaerial exposure of the formed sediment [25,29].

The layers of the Górażdże Formation are barrier deposits formed during sea transgression. The oscillatory flooding events with small-scale regressive episodes occurred there (Figure 2) [25,29,30].

The limestones are usually medium- or thick-bedded, often crumpled and micritic, including oncoids and bioclasts [29]. With the sea basin deepening, the contribution of Planolites-Palaeophycus ichnofossils increased, which were accompanied by small branched chondrites traces [25]. Therefore, the echinoid fauna contains rare elements and large spatula-shaped spines that seem to be restricted only to this formation [30].

The Dziewkowice (Terebratula) Formation represents off-barrier facies formed during the subsequent phase of transgression (Figure 2) [25,30]. The other name of this formation—Terebratula—is connected with the presence of Terebratula coquina [29]. Moreover, this formation is characterized by the occurrence of crumpled limestones and medium-bedded crinoidal packstone and grainstone [29]. The subsequent phase of transgression was characterized by moderate dysoxic conditions. It is connected with the presence of the very early pyrite-encrustations piercing the nautiloid shell [25].

The Karchowice Formation represents barrier facies formed during the sea regression (Figure 2) [25,29,30]. The fauna of this formation is basically different from that of the underlying Terebratula Beds [30]. There are thin- and medium-bedded shell-echinoderm limestones and large sponge-crinoid-coral bioherms including inter-bioherm calcirudites [29]. The fauna includes stenohaline elements that did not reach the central parts of the Germanic Basin because of a salinity barrier [30]. These faunal elements comprise hexactinellid sponges, such as Tremadictyon, Calycomorpha, Silesiaspongia, and Hexactinoderma [30].

**Figure 3:**
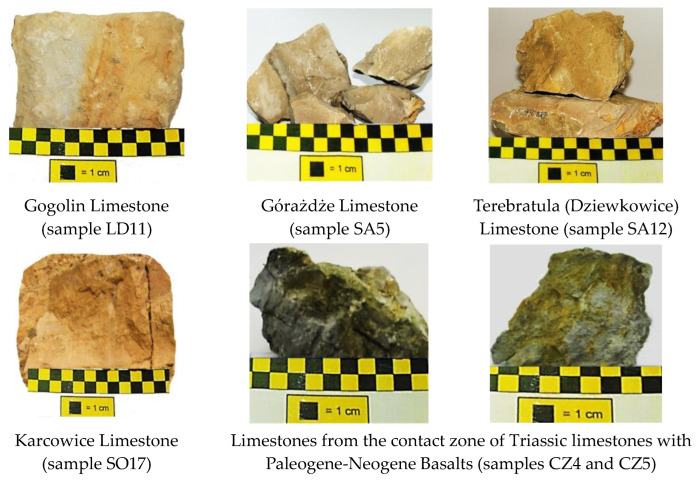
Examples of analyzed limestone samples.

The examples of limestone of four Lower Muschelkalk formations—Gogolin, Gorażdże, Terebratula (Dziewkowice), and Karchowice—and two samples of limestone from the contact zone of Triassic limestones with Paleogene-Neogene basalts are presented in Figure 3. It is possible to notice that the limestones macroscopically are different in color, which is related to the presence of admixtures, for example, iron compounds (yellow, red, brown color) or organic matter (dark grey color).

The results of previous studies indicate that the analyzed limestones are composed of the following carbonate phases: low-Mg calcite, high-Mg calcite, proto-dolomite, ordered dolomite, and huntite [1,2,3,4,5,6,7,8,9].

### 2.2. Methods

Limestone samples were tested using three analytical methods:

- Analysis of average chemical composition:
  – ICP-MS: The measurements were made by applying a ZSX Primus II Rigaku spectrometer, Rigaku, Tokyo, Japan, equipped with the 4 kW, 60 kV Rh anode and wavelength dispersion detection system.
  – Spectrometry X-ray fluorescence (XRF): The measurements were made using the wave-dispersive X-ray fluorescence spectrometer, ZSX PRIMUS RIGAKU, Rigaku, Tokyo, Japan, equipped with a rhodium X-ray tube with the possibility of smooth setting of the voltage of 20–60 kV, analytical crystals LiF, Ge and several synthetic crystals.
- Point analysis of chemical composition in a micro area:
  – Electron microprobe analysis (EPMA): The measurements were made using a JXA-8230 X-ray micro-analyzer manufactured by JOEL, Santa Monica, CA, USA. The examinations were performed on polished sections which were sputtered with a carbon coat. The analysis with the application of WDS spectrometers was carried out in micro-areas of two samples—G1 and LD11.

These three methods were used because they complement each other. The XRF method allows for the determination of the average content of macro constituents, and the ICP-MS method, due to its low detection limits, allows for the determination of the average concentration of trace elements.

The EPMA method allows for the determination of the chemical composition of a single mineral grain, which is extremely helpful in interpreting the phase composition of the sample. For the purposes of this article, it provides valuable information regarding the content of elements, the average concentration of which in the sample is traces. The samples for microprobe measurements were selected from all samples on the basis of the results of the previous ICP-MS and X-ray fluorescence analyses.

It should be noted that two different methods were used for the determination of the average concentrations of the elements. Each of these methods has a different limit of detection (LOD) and limit of quantification (LOQ) for the same element (Table 2). Due to the fact that trace elements occur in very small amounts and their concentration may be slightly different in the same limestone sample, the two different methods may result in different determinations.

Additionally, in the case of XRF, the measurement is made in a polished or polished grain mount section of the sample.

## 3. Results

### 3.1. ICP-MS

The results of measurements of the selected trace elements: Ti, Zn, Pb, Cu, Sr, Ba, Cr, Ni, Nb, Zr, Rb, Mo, Cd, and Hf, using ICP-MS were presented in Table 3, Table 4, Table 5 and Table 6 [9]. The study results show that the contents of trace elements are different in analyzed samples (Figure S1).

The higher amount of Ti (Figure S1) was measured in two limestone samples from the Karchowice Beds (samples SO17 and SO20). In limestones from the Gogolin, Górażdże, and Terebratula Beds and sample SO14 from the Karchowice Beds is lower. There is no relationship between the Pb content in the samples and their lithostratigraphy. The content of Pb (Figure S1) is generally low, below 80 ppm. The highest content of Ba (Figure S3) was determined only in one sample from the Terebratula limestones (sample S2). The amount of Sr is increased generally in samples of all formations (Figure S1). The lowest content of Sr was determined in one sample from the Terebratula Beds (sample SA12). The higher amounts of Cr, Ni, and Mo were determined in some limestone samples from the Terebratula Beds and Karchowice Beds. The amount of other trace elements—Cu, Zn (Figure S4), Rb, Nb, Cd, Hf—are definitely lower.

The content of these elements is sometimes lower than 1 ppm (Table 3) [9]. The differentiation of Zn content is observed in rocks from all the formations (Figure S1). Therefore, the high amounts of some trace elements—Ti, Cr, and Ni—were measured in the limestones from all formations.

The increased contents of Sr and Mo were also determined in limestone samples from all formations.

Lower amounts of Zr, Ba, Pb, and Cu were measured. The lowest amounts were Rb, Nb, Cd, and Hf. The test results presented in Table 3 and Figure S1 show a general tendency toward increased content of trace elements in the limestones of the Karchowice Beds apart from the amount of Ba. This may be related to the formation of these sediments during sea regression [25]. During sea regression, the area of the seabed rises up and autogenous minerals are supplied to the reservoir with the fresh water. There is also the release of trace elements from autogenous minerals during weathering and erosion. Moreover, it can be noticed that the content of some elements—especially Ti, Cr, Ni, and Mo—in the limestones of the same formation varies. This is particularly evident in the case of the limestones of the Terebratula Beds and Karchowice Beds. Probably, it can only be related to the variable concentration of the trace elements on the bottom of the sea basin during sedimentation because there were no dislocations that could affect the obtained values of the content of elements.

The results of the research show a very low content of Y, Sc, and REEs (Table 4, Figure S2). The yttrium content varies from less than 1 ppm to 3 ppm, and scandium content varies from 0 ppm to 10 ppm (Table 4, Figure S2). Some trace elements, including REE, Y, and Sc, could be affected by sedimentary processes and transferred into the clastic sedimentary record during continental erosion [31]. Yttrium could be inserted between Dy and Ho because of the similar ionic size and charge of Y and Ho [32]. However, they probably substitute Ca in carbonate crystal structures like other REEs.

Among REEs, only Ce, Nd, Sm, and Dy were determined in analyzed rocks (Table 4, Figure S3); although other REEs were also measured, they were not found. Moreover, their contents are very low, sometimes below 1 ppm. The contents of Ce, Nd, Sm, and Dy vary: from below 1 ppm to 6 ppm for Ce, from below 1 ppm to 4 ppm for Nd, from below 1 ppm to 2 ppm for Sm, and from below 1 ppm to 1 ppm for Dy.

**Figure 4:**
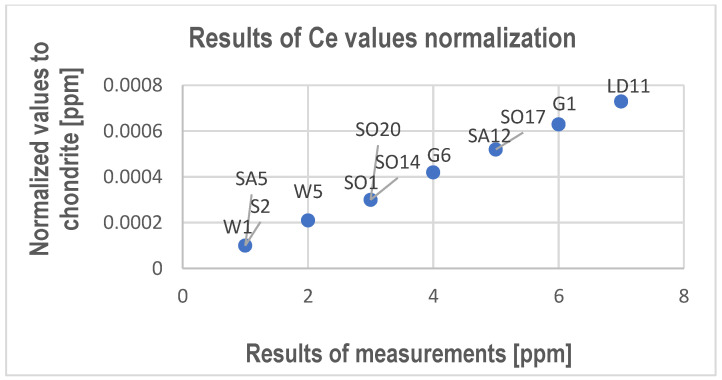

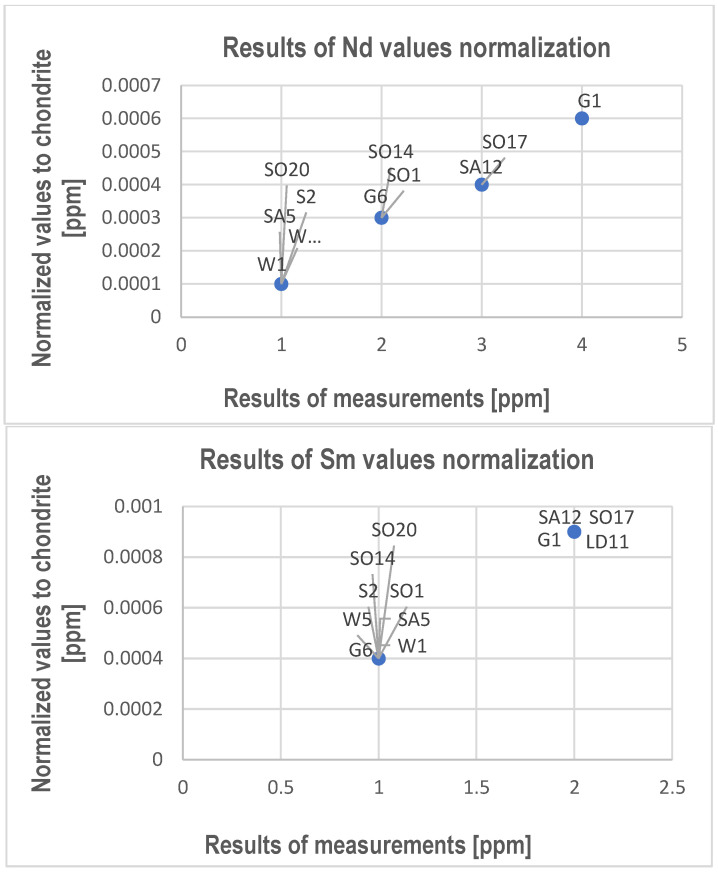
Results of Ce, Nd, and Sm normalization to chondrite according to Taylor and McLennan [33].

The test results were normalized to chondrite according to Taylor and McLennan [33], including the upper continental crust [31,34]. The results are presented in Figure 4. In the case of dysprosium, the normalized value for all samples was the same—0.0003 ppm.

According to the data of previous studies, the content of REEs in the earth-crust limestone varies from 0.2 ppm (in the case of Europium and Terbium) to 11.5 ppm (in the case of Cerium) [35,36]. Compared to previous studies, it can be concluded that the analyzed limestones are poor in REEs. Only four elements, Ce, Nd, Sm, and Dy, were determined in the limestones. However, it should be noted that the REE contents in the samples from the Gogolin Beds are higher compared to the others. Analyzing the results of normalization, it can be said that all values are below 0.001 ppm. These results indicate a significant depletion of the tested carbonate rocks in Ce, Nd, Sm, and Dy compared to earth-crust limestones [31,34,35,36]. When analyzing Ce results, it is necessary to point out first that compared with the strictly trivalent REE, cerium (Ce), like europium (Eu), is the only commonly multivalent element among the REEs [32]. The results of REE measurements indicate the occurrence of very low values of Ce, Nd, Sm, and Dy. The Ce values determined in samples G1, G6, LD11, W5, SA12, SO1, SO14, SO17, and SO20 are slightly higher than the Nd, Sm, and Dy values. This indicates a slight positive Ce anomaly in relation to other elements but definitely negative in relation to the upper continental crust [31,37]. However, the measured values of Ce, Nd, Sm, and Dy are too low to provide more detailed test results and the cerium anomaly.

### 3.2. X-ray Fluorescence (XRF)

The results of X-ray fluorescence of the selected trace elements P, S, Ti, Cr, Ni, Cu, Zn, Rb, Sr, Y, Zr, Nb, Mo, and Pb are presented in Table 5 [9] and Table 6. The test results are also presented in Figure S4.

The results of the previous research indicate that the analyzed limestones are characterized by different Ca content, from 27.34% (38.28% CaO) to 40.89% (57.20% CaO) [9]. The purest calcite contains 56.03% CaO and 43.97% CO_2_ [32]. The results of the previous studies showed that the analyzed rocks are composed of carbonate phases with different Ca and Mg contents: low-Mg calcite (pure calcite without substitution), high-Mg calcite, proto-dolomite, ordered dolomite, and huntite [2,3,4,5,6,7,8,29]. Some trace elements can substitute for calcium in these minerals.

The results of XRF show the increased contents of P, Cr, Ni, and Cu in limestones from the Karchowice Beds and that of Pb, Ti, Sr, and S (Figure S4) in limestones from all formations. The graph of Ti content, presented in Figure S4, is presented differently than the others. It is presented diagonally due to the large difference in the Ti content in the CZ3 sample compared to the other samples. The values of Zn (Figure S4), Rb, Zr, Nb, and Mo are different in the samples from all formations. Therefore, there is no relationship between these elements and lithostratigraphy. The barium was determined only in three samples of limestones from the contact zone, while Mo and Er were determined in some samples from other limestones (Table 5 and Table 6). Therefore, the obtained data indicate that the highest contents of some trace elements concentrate in some limestones from the Karchowice Beds (Table 5 and Table 6, Figure S4) in the rocks of the sea regression stage (Karchowice Beds) [9]. As mentioned earlier, in the samples from the Gogolin layers, the rocks of the initial sea transgressive stage do not show above-average content of some elements compared to other samples.

The test results show generally a higher content of elements, such as P, Ti, Zn, Zr, and Pb, in limestones from the contact zone with basalts than in limestones from other areas. In the case of the other elements, the values are comparable or slightly lower for some samples.

**Figure 5:**
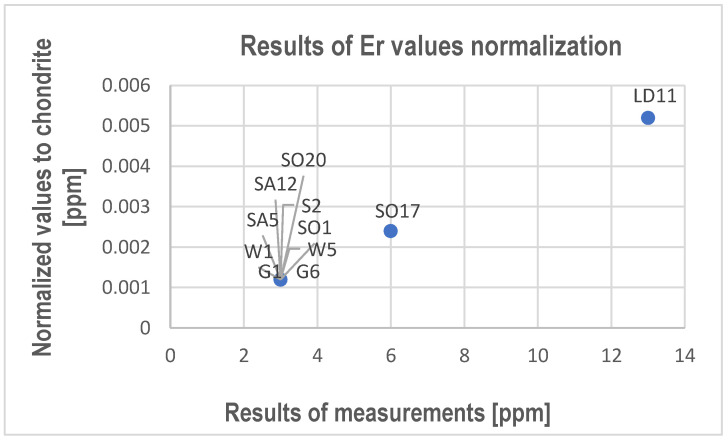
Results of Er normalization to chondrite according to Taylor and McLennan [33].

The obtained data show that it is worth paying attention to the increased content of Zn in Górażdże limestone. Since sulfur was also found in the examined limestones, it can be assumed that zinc will probably be bound in sulfides. The presence of sulfides may indicate the influence of hydrothermal processes that caused the mineralization of the limestones. The XRF allowed us to determine the content of Y, which ranges from 1 to 9 ppm (Figure S4). In three samples (Table 5 and Table 6), only erbium was found. Although other REEs were also measured, they were not found. Moreover, the Er content was very low. It ranged from 3 ppm to 13 ppm. The test results from the ICP-MS analysis were normalized to chondrite according to Taylor and McLennan [33], including the upper continental crust [31,34] (Figure 5).

These results also indicate a significant depletion of the tested carbonate rocks in Er compared to earth-crust limestones [31,34,35,36] as in the case of Ce, Nd, Sm, and Dy determined by ICP-MS analysis.

It is worth paying attention to the presence of erbium in the limestones of the Gogolin Beds and Karchowice Beds, which include the rocks of barrier facies of the initial sea transgressive stage and the rocks of barrier facies of the sea regression stage.

### 3.3. Electron Probe Microanalysis

The microprobe analysis was applied to conduct quantitative analyses in micro-areas to identify REEs, yttrium, and scandium. It was executed for two samples: G1 and LD11. During the measurements, the contents of only some rare earth elements—Ce, Nd, Sm, Gd, Dy, Y, and Sc—were determined.

#### 3.3.1. Sample G1

**Figure 6:**
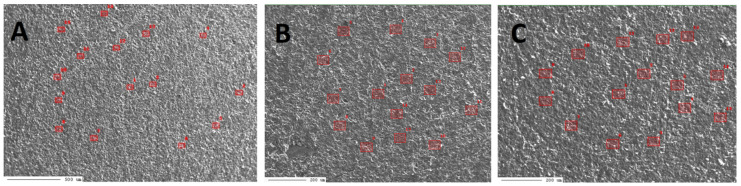
BSE image of the first micro-area (**A**), the second micro-area (**B**), and the third micro-area (**C**) of sample G1 (Gogolin Limestone from Gogolin Deposit.

In the case of sample G1, measurements were carried out in three micro-areas (Figure 6A–C, Tables S1–S6).

In the first micro-area of sample G1 (Figure 6A), the percentage content of elements was determined in 15 points. The results are presented in Tables S1 and S2.

The results of measurements executed in the first micro-area of sample G1 indicate that, in many points, Ce was determined, and in some points, Dy, Nd, Sm, and Ga were determined (Table S2). However, the Dy content, measured only in two points (Tables S3 and S6), exceeded 100 ppm. Very low amounts of Y and Sc were determined. Moreover, the Sc was determined only at one point. The percentage content of elements in the points was determined. The results are presented in Tables S3 and S4.

In micro-area 2 (Figure 6B), during the second series of measurements, also in 15 points, the percentage content of elements was determined. The results are presented in Tables S3 and S4. The test results indicate that also in micro-area 2 of the G1 sample, measurements were made within the low-magnesium calcite phase (Table S3).

The results of measurements executed in the second micro-area of sample G1 indicate that, as in micro-area 1, in many points, Ce was determined. In some points, Nd, Sm, and Ga were measured, and in only one point—point 1—Dy was determined (Table S6). However, the Dy content exceeded 100 ppm. As in micro-area 1, also in this area of sample G1, very low amounts of Y and Sc were determined. Moreover, Sc was also measured only at one point—point 6.

In micro-area 3 (Figure 6C), during the third series of measurements, the percentage content of elements was also determined in 15 points. The results are presented in Tables S7 and S9.

The results of measurements executed in the third micro-area of sample G1 indicate that, as in micro-areas 1 and 2, in many points, Ce was determined. In some points, Nd, Sm, and Ga were measured, and in only two points—points 1 and 2—Dy was determined (Table S6). However, the Dy content measured in point 1 exceeded 100 ppm. As in micro-areas 1 and 2, also in this area of sample G1, very low amounts of Y and Sc were determined. Moreover, Sc was also measured only at one point—point 6.

The results of measurements in the micro-areas of the G1 sample indicate that the content of Ce in selected points ranges from 3 to 58 ppm, Nd from 1 to 37 ppm, Sm from 1 to 40 ppm, Gd from 1 to 37 ppm, and Dy from 34 to 384 ppm. In the case of yttrium and scandium, the contents at selected points range from 4 to 21 ppm for Y, and from 1 to 2 ppm for Sc.

#### 3.3.2. Sample LD11

**Figure 7:**
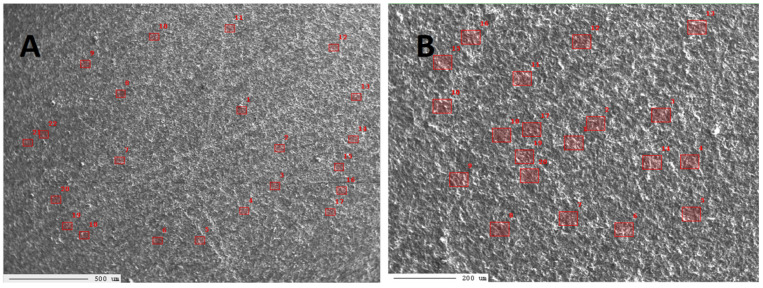
BSE image of the first micro-area (**A**) and the second micro-area (**B**) of the sample LD11 (Gogolin Limestone from Ligota Dolna Deposit).

In the case of the LD11 sample, the measurements were carried out in two micro-areas (Figure 7A,B, Tables S7–S10).

In the first micro-area of sample LD11 (Figure 7A), during the first series of measurements, the percentage content of elements was determined in 17 points. The results are presented in Tables S7 and S8. The test results indicate that in micro-area 1 of the LD11 sample, measurements were made within the low-magnesium calcite phase (Table S5).

The results of measurements executed in the first micro-area of the sample LD11 indicate that, in many points, Ce was determined, and in some points—Nd, Sm, Ga, and Dy were determined (Table S8). As in micro-areas of sample G1, very low amounts of Y and Sc were determined.

In the second micro-area of the sample LD11 (Figure 7B), during the second series of measurements, the percentage content of elements was determined in 20 points. The results are presented in Tables S9 and S10. The test results indicate that in micro-area 2 of the LD11 sample, measurements were made within the low-magnesium calcite phase (Table S9).

The results of measurements executed in the second micro-area of sample LD11 indicate that, as in micro-area 1 of sample LD11, in many points, Ce was determined. In some points, Nd, Sm, Ga, and Dy were determined (Table S10). Moreover, the Dy content measured in points 4, 10, and 18 exceeded 100 ppm. As in the micro-areas of sample G1 and the first micro-area of sample LD11, very low amounts of Y and Sc were determined.

The results of measurements in the micro-areas of the LD11 sample indicate that the content of Ce at selected points ranges from 2 to 59 ppm, Nd from 1 to 35 ppm, Sm from 1 to 35 ppm, Gd from 5 to 44 ppm, and Dy from 25 to 129 ppm. In the case of yttrium and scandium, the contents at selected points range from 1 to 22 ppm for Y and from 1 to 6 ppm for Sc.

The electron probe microanalysis (microprobe measurements) allowed us to determine in various micro-area points the contents of selected elements belonging to the LREY cerium group—cerium, neodymium, samarium; elements belonging to the MREY terbium group—dysprosium and gadolinium; and element belonging to the HREY yttrium group—erbium. Their content at selected points varies. Ce content ranges from 2 to 59 ppm, Nd from 1 to 37 ppm, Sm from 1 to 40 ppm, Gd from 1 to 44 ppm, and Dy from 25 to 384 ppm. In the case of yttrium and scandium, the contents at selected points range from 1 to 22 ppm for Y and from 1 to 6 ppm for Sc.

## 4. Discussion

The test results indicate the increased content of some of the analyzed trace elements in studied limestones [9].

Increased amounts were found in the case of S and P. Moreover, the higher contents of Sr, Cr, Ni, and Mo were measured in some samples. Much smaller amounts were determined in the case of Pb, Zn, and Cu and the lowest amounts were for Nb, Cd, Zr, Hf, and Rb.

The highest contents of Ba were determined in sample S2 and in three limestone samples—CZ3, CZ4, and CZ5—which come from the contact zone with basalts.

The test results show that the highest contents of trace elements were measured in the limestones of the Karchowice Beds, the rocks that were formed during sea regression. The contents of the studied trace elements, which were determined in the limestones of the Gogolin Beds—the rocks that were formed during the initial sea transgressive stage—and in the limestones of the Górażdże Beds and the Terebratula Beds—the rocks that were formed during sea transgression—are comparable. Therefore, the increased content of some trace elements may be related to the low sea level that took place during marine regressions [9,25] because then the area of the seabed rises up and autogenous minerals are supplied to the reservoir with the fresh water. There is also the release of trace elements from autogenous minerals during weathering and erosion. The increased contents of some trace elements determined in the limestones from the contact zone with Paleogene-Neogene basalts compared to the other limestones may be related to the influence of magma activity.

Analyzing the origin of trace elements, it can be assumed that some trace elements may appear as substitutions in clay or carbonate minerals. The results of phase analyses performed during previous studies confirmed this theory [2,4,5,9,35]. Zn, Pb, Cu, Mo, and Ni can be bound in sulfides, and Ti and Cr can be bound in oxides.

The occurrence of sulfur may confirm the presence of sulfides. These minerals could have been formed during hydrothermal processes, the same as the carbonate rocks of the Upper Silesia and Kraków areas [26,27]. Sulfur is bound in sulfides, usually Zn, Pb, and Cu sulfides, and also in sulfates. During the tests, increased amounts of P were also determined. This element can be associated with organic matter [9,33]. Sr and Ba are common elements in carbonate minerals. In some publications, the authors state that Sr could be present in skeletons of marine organisms [4,9,19,20,21,35,38,39]. The remains of marine organisms were analyzed during previous research. They were observed in limestones macroscopically and during microscopic analysis of thin sections [4,9,35]. Sr usually occurs in aragonite.

The presence of strontium in the aragonite carbonate phase is possible because Sr has a bigger ionic radius than the Ca radius [22]. Therefore, strontium enters into the aragonite structure more easily than the calcite one. This is possible because the aragonite structure is analogous to the strontianite structure. Summarizing, it should be stated that the material built of aragonite contains a greater amount of Sr than the one built of calcite [9,19,22,35]. But because aragonite is an unstable carbonate phase, just like high magnesium calcite, it is transformed into low magnesium calcite the same as high-Mg calcite during diagenetic processes. Therefore, the occurrence of strontium currently in calcite indicates that the primary carbonate material included aragonite [10,11,19,20,21,22].

Barium, similar to Sr, can be found in the skeletons of marine organisms. Because barium has a similar ionic radius to strontium, which is greater than calcium, it will substitute calcium in carbonates just like Sr [9,19,20,29,39]. That’s why, like strontium, Ba will enter into an aragonite structure more easily than into a calcite structure. Therefore, the Ba presence currently in low-Mg calcite indicates that the primary carbonate material included aragonite.

The content of Ni ranges from 17 to 18,000 ppm depending on the applied research method. This element is bound in clay minerals. The binding of Ni in the structure of clay minerals takes place during sedimentation processes. Therefore, clay minerals containing Ni were delivered to the sea basin with fresh water or were formed in this basin during diagenetic processes. Ni could also be bound in sulfides.

Zinc and lead sulfides, which contain Ni substitutions, are the products of hydrothermal processes. The content of Cd ranges from below 1 ppm to 120 ppm. Cadmium is an ingredient of Zn sulfides. The content of Zr ranges from 0 to 41 ppm. Zirconium is usually found in seawater; therefore, it can be delivered with fresh water to a basin where carbonate sedimentation takes place. It may also be bound in non-carbonate autogenic minerals (clay minerals, feldspars, and feldspathoids). The content of Hf ranges from below 1 ppm to 2 ppm. It is a very low content. Hafnium usually coexists with zircon, and in all geochemical environments, it occurs together with Zr in a dispersed form. Such low contents may indicate that Hf is substituting other elements in minerals. The content of Nb ranges from 0 to 40 ppm. Niobium can occur as an admixture in the titanium and zirconium minerals or Mn concretions [1,2,3,4,5,6,7,8,9]. The content of Cr ranges from 0 to 9400 ppm. Chromium is usually dispersed in rock-forming minerals. Usually during the weathering processes, this element is released from minerals. But just after that, Cr is quickly bound into structures of minerals that are the products of weathering, usually into structures of clay minerals [9,40]. The content of Mo ranges from 0 to 9000 ppm. Molybdenum, like Ni and Cr, is bound in clay minerals. Molybdenum, in an amount of up to 1%, may coexist with manganese concretions that are situated in the deep zones of the sea. The content of Rb ranges from 0 to below 10 ppm. This content is low. Such low contents may indicate that Rb is substituting other elements in K feldspar and feldspathoids minerals because this element coexists with potassium in K feldspar and feldspathoids [40,41,42]. Small amounts of Rb can be found in seawater [9].

Yttrium and lanthanides (REE) can be often found in carbonate rocks. Sc and REY usually substitute Ca in calcite structures and sometimes in dolomite structures [15]. In general, the content of yttrium varies in carbonate phases from 1 to 9 ppm, and scandium varies from 0 to 10 ppm. These contents are very low. Yttrium occurs in carbonates which build skeleton remains of marine organisms. It could also be delivered to carbonate mud and preserved by warm fluids mixing followed by early lithification in shallow burial marine environments during early diagenesis. ICP-MS, XRF, and electron probe microanalysis allowed us to determine that in investigated limestones only Ce, Nd, and Sm (lanthanides of the LREE group, identified by ICP-MS and by electron probe microanalysis), Ga and Dy (lanthanides of the MREY group—Dy was identified by ICP-MS and electron probe microanalysis, and Ga was identified by electron probe microanalysis) and Er (lanthanides of the HREE group, identified by XRF). Although the other REEs were also measured, they were not found. Moreover, the results indicate a very low content of REEs. Moreover, the contents of selected elements belonging to the LREY cerium group (cerium, neodymium, samarium), the MREY terbium group (dysprosium and gadolinium), and the HREY yttrium group (erbium) were determined by measurement in points using electron probe microanalysis. The contents measured at selected points (for Ce from 2 to 59 ppm, for Nd from 1 to 37 ppm, for Sm from 1 to 40 ppm, for Gd from 1 to 44 ppm, and for Dy from 25 to 384 ppm) are usually higher than those measured by ICP-MS. In the case of yttrium and scandium, the contents are also very low (for Y, 1 to 22 ppm, and for Sc, 0 to 10 ppm).

The results of the research show a low content of analyzed trace elements. This indicates the high purity of the tested limestones. The contents of the identified REEs—Ce, Nd, Sm, Er, Dy, Ga, Y, and Sc—are very low. Although they have been determined in the studied limestones, with even higher values measured in points using electron probe microanalysis, the average content measured in rock samples is too low to be worth extracting them from limestones.

## 5. Conclusions

One of the most important results to come from the investigation of the Triassic (Muschelkalk) limestones of the area of Opole Silesia in Poland is the identification of some trace elements and some REY elements and Sc. The principal results can be summarized as follows:

1. The results of the research showed that, in the examined limestones, an increased content of some analyzed elements was measured. Increased amounts were determined in the case of S, Sr, Ba, Cr, Ni, and Mo, and in some samples of Karchowice limestones and limestones from the contact zone with Paleogene-Neogene basalts. Lower contents were determined for Pb, Zn, and Cu and were the lowest for Nb, Cd, Zr, Y, Hf, and Rb.
2. The increased content of trace elements was determined in the limestones from the Karchowice Beds. The amounts of trace elements determined in the rocks from the Gogolin Beds, from the Górażdże Beds, and from the Terebratula Beds are comparable. The higher amount of some trace elements determined in the limestones of the Karchowice Beds may be related to the low sea level during marine regressions. The increased contents of some trace elements determined in the limestones from the contact zone with basalts may be related to the influence of magma activity.
3. Trace elements like Zn, Pb, Cu, Mo, and Ni are probably bound in sulfides. Ti and Cr are probably bound in oxides.
4. Sulfur is an ingredient of Fe, Zn, Pb, and Cu sulfides or is bound in sulfates. P is a component of organic matter.
5. Sr and Ba are common elements in some carbonate minerals. The presence of Sr and Ba indicates that primary calcium carbonate material included aragonite. Aragonite was transformed into low-magnesium calcite during the diagenetic processes; therefore, today Sr and Ba are present in calcite.
6. Ni is bound in clay minerals or in sulfides. Cadmium is an ingredient of Zn sulfide. Zr can be delivered with fresh water to a basin where carbonate sedimentation takes place. It may also be bound in non-carbonate autogenic minerals. Hf usually coexists with Zr. Nb can occur as an admixture in the Ti and Zr minerals or Mn concretions.
7. Chromium is dispersed in rock-forming minerals. Mo occurs in clay minerals. In content up to 0.1%, it may coexist with Mn concretions that are situated in the deep zones of the sea. Rb coexists with K in feldspars or feldspathoids. Small amounts of Rb could be found in seawater.
8. REY and Sc substitute Ca usually in calcite and sometimes in dolomite. The tests allowed us to determine only Ce, Nd, Sm, Dy, Ga, Er, Y, and Sc.
9. Though they have been determined in the studied limestones, with even higher values measured in points using electron probe microanalysis, the average content measured in rock samples is too low to be possible to recover these elements from limestone, and the products of the processing of these rocks do not have a harmful impact on the environment.

## Figures and Tables

**Table 1:** List of locations where the samples presented in the paper were collected.

Points Marked on the Map in Figure 1	Location Name	Geographical Coordinates (GPS Data)
3	Szymiszów	50°32′11″ N 18°13′35″ E
4	Saint Anne Mountain (amphitheater)	50°27′18″ N 18°9′38″ E
4	Saint Anne Mountain (nephelinite quarry)	50°27′12″ N 18°9′59″ E
4	Wysoka (near Saint Anne Mountain)	50°28′32″ N 18°10′12″ E
5	Ligota Dolna and Kamienna	50°29′14″ N 18°7′27″ E
12	Strzelce Opolskie	50°31′53″ N 18°18′41″ E
13	Gogolin	50°30′10″ N 18°1′59″ E

**Table 2:** Limit of detection (LOD) and limit of quantification (LOQ) of XRF and ICP-MS methods.

No.	Element	ICP-MS Method		XRF Method	
		LOD [ppm]	LOQ [ppm]	LOD [ppm]	LOQ [ppm]
1	Ti	3	4	19	22
2	Cr	3	4	10	12
3	Ni	15	16	6	8
4	Cu	10	12	5	6
5	Zn	15	16	5	6
6	Rb	1	4	3	4
7	Sr	3	4	4	5
8	Zr	1	2	19	21
9	Nb	1	2	7	9
10	Mo	3	4	6	8
11	Cd	1	2	-	-
12	Ba	3	4	-	-
13	Hf	1	2	-	-
14	Pb	3	4	8	9
15	Ce	1	2	-	-
16	Nd	1	2	-	-
17	Sm	1	2	-	-
18	Dy	1	2	-	-
19	Y	1	2	3	4
20	Sc	1	2	-	-
21	Er	1	2	3	4

**Table 3:** Results of ICP-MS of trace elements in the samples from Gogolin and Górażdże Beds [9]—samples G1—SA5 and the samples from Terebratula and Karchowice Beds [9]—samples SA12—SO20.

No.	Element	Sample Numbers (Element Content in ppm)											
		G1	G6	LD11	W1	W5	SA5	SA12	S2	SO1	SO14	SO17	SO20
1	Ti	250	100	330	90	110	100	270	100	110	190	380	1700
2	Cr	320	260	200	260	460	210	1200	1300	260	210	220	9400
3	Ni	350	340	330	380	750	340	310	1600	400	350	360	18,000
4	Cu	<10	<10	290	<10	30	<10	10	10	10	30	60	60
5	Zn	20	110	30	50	70	110	30	70	20	40	50	240
6	Rb	<1	<1	<1	<1	<1	<1	<1	<1	<1	<1	<1	<1
7	Sr	260	120	450	240	370	240	32	230	360	210	540	160
8	Zr	20	10	10	10	20	<1	10	30	<10	<10	20	10
9	Nb	40	<1	<1	<1	<1	<1	10	10	10	10	10	40
10	Mo	120	120	120	130	210	140	120	1100	220	170	160	9000
11	Cd	<1	<1	1	1	1	<1	<1	2	<1	<1	<1	120
12	Ba	24	15	29	10	20	16	28	260	20	20	34	35
13	Hf	2	<1	<1	2	2	1	<1	1	<1	<1	1	<1
14	Pb	25	9	79	24	39	20	27	22	10	20	20	62

**Table 4:** Results of ICP-MS of REY in analyzed samples.

Element	Sample Numbers (Element Content in ppm)											
	G1	G6	LD11	W1	W5	SA5	SA12	S2	SO1	SO14	SO17	SO20
Ce	6	4	7	1	2	<1	5	1	3	3	5	3
Nd	4	2	4	1	1	<1	3	<1	2	2	3	1
Sm	2	1	2	1	1	<1	2	<1	<1	<1	2	<1
Dy	1	<1	1	<1	<1	<1	<1	<1	<1	<1	<1	<1
Ʃ REE	~14	~7	~14	~3	~4	<1	~10	~1	~5	~5	~10	~4
Y	2	2	2	1	1	<1	2	1	3	3	3	2
Sc	<10	<10	<10	<10	<10	<10	<10	<10	10	10	<10	<10

**Table 5:** Results of XRF of trace elements in the samples from the Gogolin and Górażdże Beds [9]—samples G1—SA5 and in the samples from the Terebratula and Karchowice Beds [9]—samples SA12—SO20.

No.	Element	Element Content in ppm											
		G1	G6	LD11	W1	W5	SA5	SA12	S2	SO1	SO14	SO17	SO20
1	P	48	41	73	39	56	38	60	93	54	201	331	150
2	Ti	114	25	235	31	60	24	173	44	65	155	293	87
3	Cr	320	260	200	260	460	210	13	20	15	9076	40	14
4	Ni	20	17	17	22	20	23	20	23	21	1304	27	24
5	Cu	23	21	20	23	19	17	20	17	21	731	25	21
6	Zn	16	15	25	44	50	90	10	54	19	66	47	44
7	Rb	4	<3	7	<3	<3	<3	6	<3	<3	<3	5	5
8	Sr	436	185	676	395	517	471	533	234	504	651	675	230
9	S	473	235	462	221	179	436	280	294	292	1854	564	328
10	Y	5	4	2	3	3	1	4	1	2	9	3	1
11	Zr	<3	3	<3	5	5	<3	-	-	-	-	-	-
12	Nb	-	-	-	-	-	-	<7	<7	<7	13	<7	<7
13	Pb	<8	<8	58	<8	33	22	<8	18	<8	<8	19	47
14	Mo	-	-	-	-	-	-	<6	<6	<6	343	<6	0
15	Er	<3	<3	13	<3	<3	<3	<3	<3	<3	<3	6	3

**Table 6:** Results of XRF of trace elements in the samples from the contact zone between Triassic limestones and Paleogene-Neogene basalts.

No.	Element	Element Content in ppm				
		CZ1	CZ2	CZ3	CZ4	CZ5
1	P	291	194	1024	72	1182
2	Ti	357	295	3056	71	404
3	Cr	39	<10	78	<10	211
4	Ni	30	33	56	21	39
5	Cu	50	38	40	47	35
6	Zn	213	102	167	51	416
7	Rb	5	<3	<3	<3	<3
8	Sr	250	245	108	203	228
9	Ba	<65	<65	114	192	197
10	S	337	241	220	146	224
11	Y	<3	<3	9	<3	5
12	Zr	29	20	41	<3	25
13	Nb	<7	<7	18	<7	<7
14	Pb	103	29	29	81	80

## Data Availability

The original contributions presented in the study are included in the article/Supplementary Materials, further inquiries can be directed to the corresponding author.

## Supplementary Materials

The following supporting information can be downloaded at: https://www.mdpi.com/article/10.3390/ma17153668/s1, Figure S1. Variability of Ti, Pb, Ba, Sr, Zn content in studied limestone samples, based on ICP-MS spectrometry. Figure S2. Variability of Zn content in studied limestone samples, based on ICP-MS spectrometry. Figure S3. Variability of Y, Ce, Nd, Sm content in studied limestone samples, based on ICP-MS spectrometry. Figure S4. Variability of Ti, Sr, Zn, S, Y content in studied limestone samples, based on X-ray fluorescence (XRF). Table S1. Results of microprobe measurements of main elements in the first micro-area of the sample G1 (%wt). Table S2. Results of microprobe measurements of Ce, Nd, Sm, Dy, Gd, Y and Sc in the first micro-area of the G1 (ppm). Table S3. Results of microprobe measurements of main elements in the second micro-area of the sample G1 (%wt). Table S4. Results of microprobe measurements of Ce, Nd, Sm, Dy, Gd, Y and Sc in the second micro-area of the G1 (ppm). Table S5. Results of microprobe measurements of main elements in the third micro-area of the sample G1 (%wt). Table S6. Results of microprobe measurements of Ce, Nd, Sm, Dy, Gd, Y and Sc in the third micro-area of the G1 (ppm). Table S7. Results of microprobe measurements of main elements in the first micro-area of the sample LD11 (%wt). Table S8. Results of microprobe measurements of Ce, Nd, Sm, Dy, Gd, Y and Sc in the first micro-area of the LD11 (ppm). Table S9. Results of microprobe measurements of main elements in the second micro-area of the sample LD11 (%wt). Table S10. Results of microprobe measurements of Ce, Nd, Sm, Dy, Gd, Y and Sc in the second micro-area of the LD11 (ppm).
