# Supplementary material for: Middle Triassic Limestones as a Source of Trace Elements and REY
Source: Materials (Basel). 2024 Jul 25;17(15):3668. doi: 10.3390/ma17153668 (PMC11312929; doi:10.3390/ma17153668)
Supplement: Supplementary file 1 [file materials-17-03668-s001.zip › materials-3068748-supplementary.pdf]

materials

MDPI

## Supplementary Materials

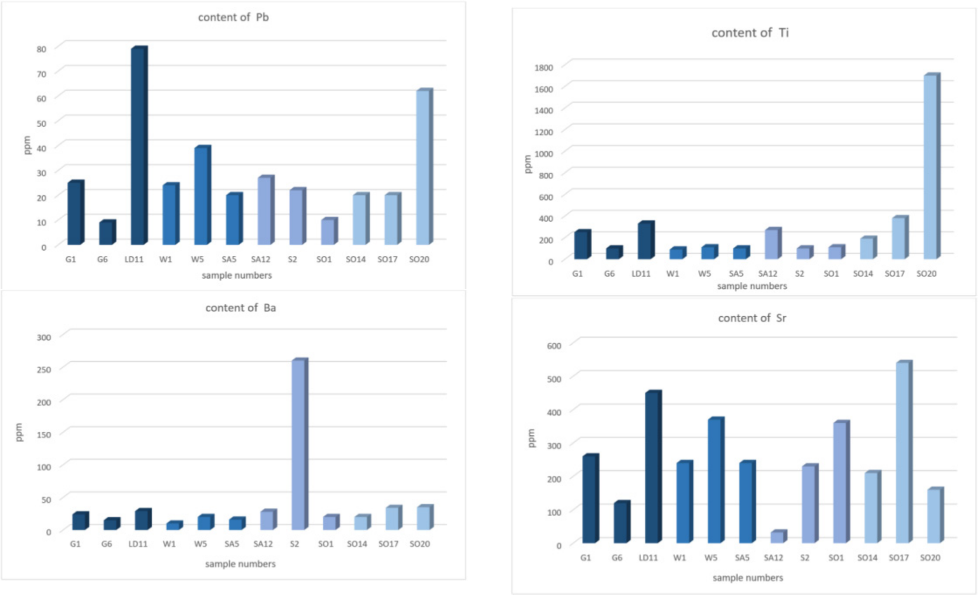


**Figure S1.** Variability of Ti, Pb, Ba, Sr, Zn content in studied limestone samples, based on ICP-MS spectrometry.

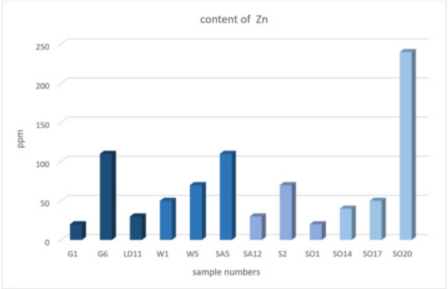


**Figure S2.** Variability of Zn content in studied limestone samples, based on ICP-MS spectrometry.

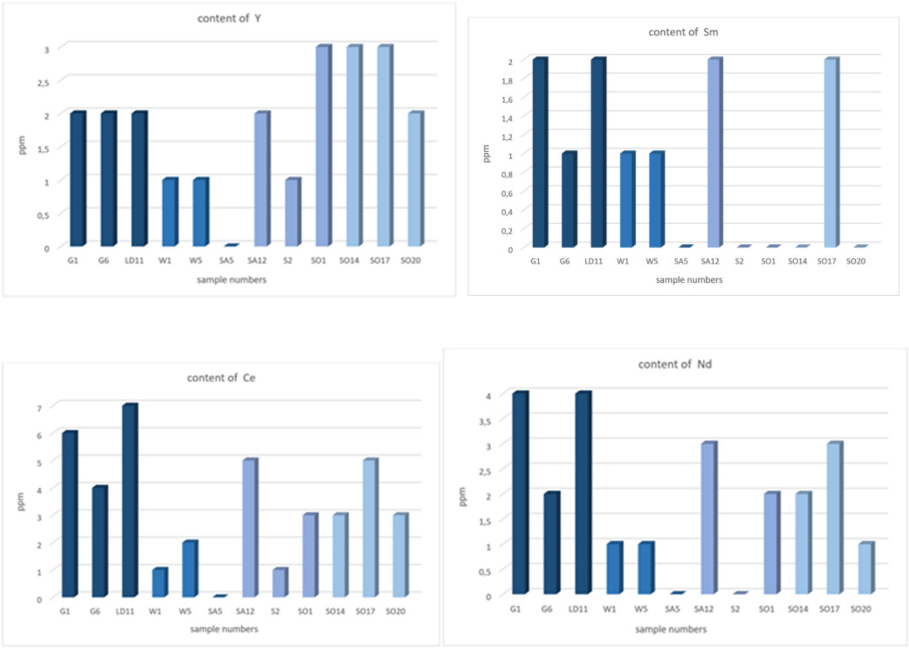


**Figure S3.** Variability of Y, Ce, Nd, Sm content in studied limestone samples, based on ICP-MS spectrometry.

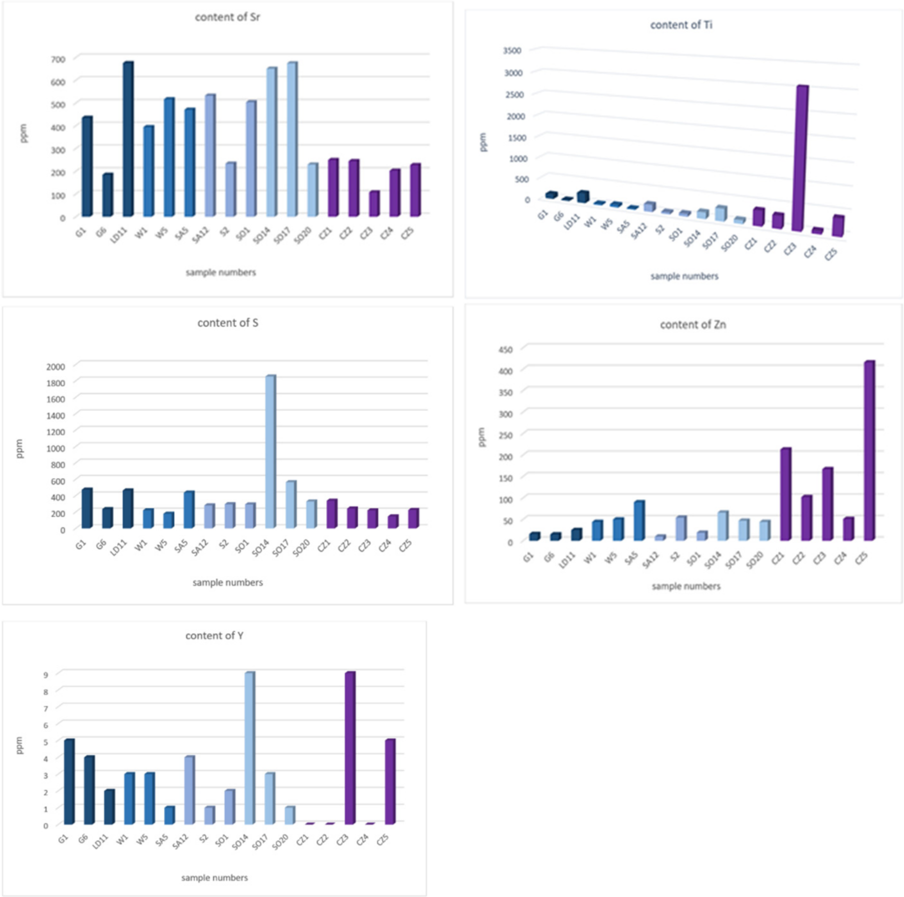


**Figure S4.** Variability of Ti, Sr, Zn, S, Y content in studied limestone samples, based on X-ray fluorescence (XRF).

**Table S1.** Results of microprobe measurements of main elements in the first micro-area of the sample G1 (%wt).

| Point No. | C | O | Al | Mg | Na | Ca | Fe | K | Si |
|---|---|---|---|---|---|---|---|---|---|
| 1 | 10.73 | 52.50 | 0.01 | 0.05 | 0.05 | 34.19 | 1.71 | 0.04 | 0.72 |
| 2 | 10.30 | 52.08 | 0.01 | 0.05 | 0.01 | 34.82 | 1.93 | 0.07 | 0.73 |
| 3 | 10.30 | 51.93 | 0.22 | 0.51 | 0.22 | 33.37 | 2.53 | 0.05 | 0.87 |
| 4 | 10.99 | 48.56 | 0.30 | 0.61 | 0.36 | 34.33 | 3.52 | 0.03 | 1.30 |
| 5 | 10.54 | 52.43 | 0.02 | 0.08 | 0.04 | 33.30 | 2.55 | 0.04 | 1.00 |
| 6 | 11.46 | 48.77 | 0.34 | 0.40 | 0.16 | 36.29 | 1.76 | 0.08 | 0.74 |
| 7 | 10.35 | 52.20 | 0.01 | 0.08 | 0.04 | 33.95 | 2.48 | 0.02 | 0.87 |
| 8 | 10.96 | 51.93 | 0.27 | 0.59 | 0.31 | 32.41 | 2.43 | 0.05 | 1.05 |
| 9 | 11.51 | 48.51 | 0.32 | 0.68 | 0.28 | 34.37 | 2.98 | 0.05 | 1.30 |
| 10 | 11.07 | 51.47 | 0.22 | 0.42 | 0.19 | 34.21 | 1.83 | 0.03 | 0.56 |
| 11 | 10.93 | 53.21 | 6.75 | 0.44 | 0.18 | 19.71 | 1.68 | 0.40 | 6.70 |
| 12 | 10.70 | 51.60 | 0.24 | 0.60 | 0.28 | 32.85 | 2.60 | 0.03 | 1.10 |
| 13 | 12.54 | 44.94 | 0.62 | 0.46 | 0.14 | 38.64 | 1.56 | 0.10 | 1.00 |
| 14 | 11.35 | 47.56 | 0.36 | 0.74 | 0.34 | 34.88 | 3.32 | 0.08 | 1.37 |
| 15 | 12.08 | 47.13 | 0.25 | 0.60 | 0.27 | 36.21 | 2.38 | 0.06 | 1.02 |
| Min. | 10.25 | 44.92 | 0.01 | 0.05 | 0.01 | 19.70 | 1.56 | 0.02 | 0.56 |
| Max. | 12.54 | 53.21 | 6.75 | 0.74 | 0.36 | 38.64 | 3.52 | 0.40 | 6.70 |
| Average | 11.03 | 50.28 | 0.6 | 0.42 | 0.19 | 33.57 | 2.35 | 0.08 | 1.36 |

**Table S2.** Results of microprobe measurements of Ce, Nd, Sm, Dy, Gd, Y and Sc in the first micro-area of the G1 (ppm).

| Point No. | Y | Sc | Ce | Dy | Nd | Sm | Gd |
|---|---|---|---|---|---|---|---|
| 1 | - | - | 16 | - | - | 6 | - |
| 2 | - | - | 19 | - | - | 12 | - |
| 3 | - | - | 11 | - | - | 14 | 14 |
| 4 | - | - | 55 | - | - | - | 37 |
| 5 | 5 | - | 30 | - | 5 | - | - |
| 6 | - | - | 9 | - | - | - | - |
| 7 | - | - | 7 | - | - | - | - |
| 8 | - | 4 | 37 | - | 13 | - | - |
| 9 | 12 | - | 18 | 384 | - | 30 | - |
| 10 | - | - | - | - | 32 | - | - |
| 11 | - | - | 6 | - | - | - | 12 |
| 12 | - | - | - | 191 | 4 | - | - |
| 13 | 8 | - | - | - | 7 | - | - |
| 14 | 5 | - | 49 | - | - | - | 20 |
| 15 | - | - | 19 | - | 4 | 12 | - |
| Min. | - | - | - | - | - | - | - |
| Max. | 12 | 4 | 55 | 384 | 32 | 30 | 37 |

**Table S3.** Results of microprobe measurements of main elements in the second micro-area of the sample G1 (%wt).

| Point No. | C | O | Al | Mg | Na | Ca | Fe | K | Si |
|---|---|---|---|---|---|---|---|---|---|
| 1 | 10.97 | 51.13 | 0.30 | 0.62 | 0.34 | 32.53 | 2.82 | 0.07 | 1.22 |
| 2 | 10.62 | 51.35 | 0.36 | 0.60 | 0.37 | 32.08 | 2.93 | 0.11 | 1.58 |
| 3 | 11.27 | 50.71 | 0.40 | 0.61 | 0.30 | 31.91 | 2.75 | 0.10 | 1.95 |
| 4 | 10.93 | 51.63 | 0.21 | 0.55 | 0.22 | 32.95 | 2.53 | 0.04 | 0.94 |
| 5 | 10.83 | 51.36 | 0.24 | 0.52 | 0.24 | 33.21 | 2.57 | 0.03 | 1.00 |
| 6 | 10.87 | 51.40 | 0.23 | 0.52 | 0.20 | 33.54 | 2.20 | 0.08 | 0.96 |
| 7 | 10.94 | 51.52 | 0.22 | 0.50 | 0.26 | 33.24 | 2.30 | 0.03 | 0.99 |
| 8 | 12.43 | 45.23 | 0.23 | 0.58 | 0.40 | 37.26 | 2.50 | 0.05 | 1.32 |
| 9 | 12.11 | 45.92 | 0.22 | 0.53 | 0.20 | 38.08 | 2.01 | 0.06 | 0.87 |
| 10 | 12.44 | 45.88 | 0.03 | 0.08 | 0.04 | 38.64 | 1.92 | 0.07 | 0.90 |
| 11 | 11.97 | 47.03 | 0.04 | 0.09 | 0.04 | 37.24 | 2.46 | 0.06 | 1.07 |
| 12 | 12.98 | 42.26 | 0.03 | 0.11 | 0.04 | 40.41 | 2.90 | 0.05 | 1.22 |
| 13 | 10.78 | 51.75 | 0.27 | 0.63 | 0.31 | 32.40 | 2.70 | 0.07 | 1.09 |
| 14 | 10.96 | 50.76 | 0.22 | 0.54 | 0.35 | 33.53 | 2.49 | 0.03 | 1.12 |
| 15 | 11.08 | 51.28 | 0.25 | 0.51 | 0.31 | 32.50 | 2.90 | 0.05 | 1.12 |
| Minimum | 10.55 | 42.17 | 0.03 | 0.08 | 0.04 | 31.91 | 1.92 | 0.03 | 0.87 |
| Maximum | 12.98 | 51.75 | 0.40 | 0.63 | 0.40 | 40.41 | 2.93 | 0.11 | 1.95 |
| Average | 11.39 | 49.26 | 0.22 | 0.47 | 0.24 | 34.63 | 2.53 | 0.06 | 1.16 |

**Table S4.** Results of microprobe measurements of Ce, Nd, Sm, Dy, Gd, Y and Sc in the second micro-area of the G1 (ppm).

| Point No. | Y | Sc | Dy | Ce | Nd | Sm | Gd |
|---|---|---|---|---|---|---|---|
| 1 | - | - | 197 | - | - | - | - |
| 2 | 4 | - | - | 19 | 33 | 17 | - |
| 3 | - | - | - | 5 | 37 | - | 7 |
| 4 | - | - | - | 40 | - | 12 | - |
| 5 | - | - | - | - | 10 | 2 | 14 |
| 6 | - | 1 | - | - | - | - | - |
| 7 | - | - | - | 3 | - | - | - |
| 8 | - | - | - | 9 | 6 | 0 | 1 |
| 9 | 21 | - | - | 41 | 0 | 0 | 10 |
| 10 | - | - | - | 31 | 0 | 1 | 22 |
| 11 | - | - | - | 4 | - | - | - |
| 12 | 21 | - | - | - | 22 | 40 | - |
| 13 | - | - | - | 28 | 1 | 4 | - |
| 14 | - | - | - | 15 | - | - | - |
| 15 | - | - | - | 39 | - | 17 | - |
| Minimum | - | - | - | - | - | - | - |
| Maximum | 21 | 1 | 197 | 41 | 37 | 40 | 22 |

**Table S5.** Results of microprobe measurements of main elements in the third micro-area of the sample G1 (%wt).

| Point No. | C | O | Al | Mg | Na | Ca | Fe | K | Si |
|---|---|---|---|---|---|---|---|---|---|
| 1 | 11.10 | 50.93 | 0.25 | 0.59 | 0.32 | 33.17 | 2.46 | 0.06 | 1.12 |
| 2 | 10.56 | 51.34 | 0.31 | 0.63 | 0.29 | 32.73 | 2.82 | 0.06 | 1.19 |
| 3 | 10.98 | 51.86 | 0.34 | 0.60 | 0.27 | 32.58 | 2.07 | 0.06 | 1.24 |
| 4 | 11.06 | 50.62 | 0.81 | 0.69 | 0.32 | 31.71 | 2.69 | 0.30 | 1.80 |
| 5 | 10.85 | 50.79 | 0.24 | 0.62 | 0.30 | 33.39 | 2.59 | 0.05 | 1.17 |
| 6 | 10.84 | 51.27 | 0.42 | 0.57 | 0.29 | 32.59 | 2.57 | 0.10 | 1.35 |
| 7 | 10.92 | 50.91 | 0.24 | 0.53 | 0.30 | 34.19 | 1.81 | 0.06 | 1.04 |
| 8 | 11.15 | 50.37 | 0.25 | 0.64 | 0.37 | 33.44 | 2.46 | 0.05 | 1.27 |
| 9 | 10.87 | 50.95 | 0.25 | 0.59 | 0.33 | 33.22 | 2.54 | 0.05 | 1.20 |
| 10 | 11.00 | 50.95 | 0.34 | 0.64 | 0.35 | 30.94 | 3.12 | 0.11 | 2.55 |
| 11 | 10.98 | 50.71 | 0.34 | 0.59 | 0.30 | 33.41 | 2.31 | 0.09 | 1.27 |
| 12 | 11.03 | 51.19 | 1.36 | 0.54 | 0.24 | 30.19 | 2.35 | 0.10 | 3.00 |
| 13 | 10.82 | 51.20 | 0.27 | 0.59 | 0.30 | 32.52 | 2.97 | 0.06 | 1.27 |
| 14 | 10.97 | 50.91 | 0.73 | 0.53 | 0.29 | 30.90 | 2.61 | 0.51 | 2.55 |
| 15 | 10.75 | 51.03 | 0.32 | 0.58 | 0.27 | 34.02 | 1.86 | 0.09 | 1.08 |
| Min. | 10.56 | 50.36 | 0.24 | 0.53 | 0.24 | 30.19 | 1.81 | 0.05 | 1.04 |
| Max. | 11.15 | 51.86 | 1.36 | 0.69 | 0.37 | 34.19 | 3.12 | 0.51 | 3.00 |
| Average | 10.90 | 50.99 | 0.43 | 0.60 | 0.30 | 32.60 | 2.48 | 0.12 | 1.54 |

**Table S6.** Results of microprobe measurements of Ce, Nd, Sm, Dy, Gd, Y and Sc in the third micro-area of the G1 (ppm).

| Point No. | Y | Sc | Dy | Ce | Nd | Sm |
|---|---|---|---|---|---|---|
| 1 | - | - | 147 | 23 | - | - |
| 2 | - | - | 34 | - | 17 | 26 |
| 3 | - | - | - | 58 | 12 | - |
| 4 | - | - | - | 24 | 11 | 6 |
| 5 | - | - | - | 5 | - | - |
| 6 | - | 2 | - | - | 6 | - |
| 7 | 10 | - | - | 30 | 3 | - |
| 8 | - | - | - | 15 | - | - |
| 9 | - | - | - | 34 | - | 9 |
| 10 | - | - | - | 18 | 20 | - |
| 11 | - | - | - | - | 10 | - |
| 12 | - | - | - | - | 8 | - |
| 13 | 10 | - | - | 6 | - | 20 |
| 14 | - | - | - | 25 | - | - |
| 15 | - | - | - | 3 | - | - |
| Min. | - | - | - | - | - | - |
| Max. | 10 | 2 | 147 | 58 | 20 | 26 |

**Table S7.** Results of microprobe measurements of main elements in the first micro-area of the sample LD11 (%wt).

| Point No. | C | O | Al | Mg | Na | Ca | Fe | K | Si |
|---|---|---|---|---|---|---|---|---|---|
| 1 | 9.30 | 53.25 | 0.59 | 0.75 | 0.37 | 29.87 | 3.14 | 0.08 | 2.65 |
| 2 | 8.75 | 53.79 | 0.46 | 0.70 | 0.25 | 31.80 | 2.63 | 0.09 | 1.53 |
| 3 | 9.20 | 53.60 | 0.39 | 0.66 | 0.20 | 30.30 | 1.91 | 0.07 | 3.67 |
| 4 | 9.01 | 53.33 | 0.48 | 0.70 | 0.31 | 30.94 | 2.93 | 0.08 | 2.22 |
| 5 | 9.16 | 53.45 | 0.33 | 0.60 | 0.26 | 33.10 | 1.74 | 0.07 | 1.29 |
| 6 | 8.91 | 53.85 | 0.77 | 0.68 | 0.31 | 29.79 | 2.79 | 0.18 | 2.72 |
| 7 | 9.14 | 52.32 | 0.54 | 0.68 | 0.29 | 32.37 | 2.74 | 0.12 | 1.80 |
| 8 | 8.99 | 53.40 | 0.70 | 0.73 | 0.33 | 30.10 | 2.95 | 0.10 | 2.70 |
| 9 | 8.81 | 53.35 | 0.53 | 0.71 | 0.39 | 30.78 | 2.89 | 0.11 | 2.43 |
| 10 | 9.02 | 53.19 | 0.48 | 0.75 | 0.38 | 30.51 | 3.16 | 0.08 | 2.43 |
| 11 | 9.10 | 53.13 | 0.49 | 0.67 | 0.34 | 31.69 | 2.38 | 0.09 | 2.11 |
| 12 | 8.59 | 53.27 | 0.50 | 0.72 | 0.41 | 30.74 | 3.07 | 0.11 | 2.59 |
| 13 | 8.70 | 53.41 | 0.47 | 0.71 | 0.36 | 31.18 | 2.88 | 0.11 | 2.18 |
| 14 | 9.23 | 53.01 | 0.41 | 0.65 | 0.34 | 31.90 | 2.32 | 0.06 | 2.08 |
| 15 | 9.01 | 53.31 | 0.48 | 0.67 | 0.30 | 30.82 | 3.02 | 0.10 | 2.29 |
| 16 | 9.17 | 53.48 | 0.54 | 0.63 | 0.35 | 30.95 | 2.68 | 0.12 | 2.08 |
| 17 | 9.11 | 52.98 | 0.40 | 0.71 | 0.31 | 31.13 | 3.05 | 0.07 | 2.24 |
| 18 | 8.80 | 53.54 | 0.49 | 0.77 | 0.28 | 31.33 | 2.84 | 0.11 | 1.84 |
| 19 | 8.53 | 54.35 | 0.05 | 0.12 | 0.06 | 31.17 | 3.06 | 0.10 | 2.56 |
| 20 | 9.65 | 50.14 | 0.50 | 0.80 | 0.37 | 33.16 | 2.85 | 0.11 | 2.42 |
| 21 | 8.53 | 53.91 | 0.56 | 0.76 | 0.45 | 28.74 | 3.64 | 0.09 | 3.32 |
| 22 | 8.55 | 54.55 | 0.08 | 0.12 | 0.05 | 31.26 | 2.86 | 0.11 | 2.42 |
| Min. | 8.49 | 50.14 | 0.05 | 0.11 | 0.05 | 28.74 | 1.74 | 0.06 | 1.29 |
| Max. | 9.65 | 54.55 | 0.77 | 0.80 | 0.45 | 33.16 | 3.64 | 0.18 | 3.67 |
| Average | 8.92 | 53.30 | 0.47 | 0.65 | 0.31 | 31.08 | 2.80 | 0.10 | 2.34 |

**Table S8.** Results of microprobe measurements of Ce, Nd, Sm, Dy, Gd, Y and Sc in the first micro-area of the LD11 (ppm).

| Point No. | Y | Sc | Dy | Ce | Nd | Sm | Gd |
|---|---|---|---|---|---|---|---|
| 1 | 22 | 1 | 98 | 0 | 20 | - | 30 |
| 2 | - | - | 45 | 15 | - | - | - |
| 3 | - | - | - | 24 | - | - | - |
| 4 | - | - | - | - | - | - | - |
| 5 | - | - | - | 16 | - | 2 | - |
| 6 | - | - | - | 9 | 10 | 9 | - |
| 7 | - | - | - | 56 | 21 | 17 | 12 |
| 8 | - | - | - | 0 | 4 | 7 | - |
| 9 | - | 2 | 40 | 2 | - | - | - |
| 10 | - | - | - | 20 | - | - | 5 |
| 11 | - | - | 25 | 28 | - | - | 17 |
| 12 | - | - | - | 5 | - | 6 | - |
| 13 | - | 3 | - | 15 | - | 8 | - |
| 14 | 1 | 6 | - | 0 | - | - | - |
| 15 | - | - | - | 16 | 12 | 3 | - |
| 16 | - | - | - | 6 | 35 | 27 | - |
| 17 | 1 | - | - | 11 | 16 | - | - |
| 18 | - | - | - | 18 | - | 35 | - |
| 19 | - | - | - | 34 | - | - | 6 |
| 20 | - | - | - | 0 | - | - | 20 |
| 21 | - | - | - | 11 | - | - | - |
| 22 | - | - | - | 52 | - | 10 | - |
| Min. | - | - | - | 0 | - | - | - |
| Max. | 22 | 6 | 98 | 56 | 35 | 35 | 30 |

**Table S9.** Results of microprobe measurements of main elements in the second micro-area of the sample LD11 (%wt).

| Point No. | C | O | Al | Mg | Na | Ca | Fe | K | Si |
|---|---|---|---|---|---|---|---|---|---|
| 1 | 9.75 | 51.95 | 0.76 | 0.75 | 0.40 | 29.62 | 3.03 | 0.51 | 3.23 |
| 2 | 9.69 | 52.05 | 0.59 | 0.73 | 0.43 | 30.69 | 3.13 | 0.14 | 2.55 |
| 3 | 9.58 | 52.87 | 0.43 | 0.72 | 0.45 | 31.55 | 2.76 | 0.08 | 1.56 |
| 4 | 9.47 | 52.13 | 0.57 | 0.78 | 0.51 | 30.55 | 2.97 | 0.12 | 2.90 |
| 5 | 9.67 | 52.20 | 0.64 | 0.72 | 0.38 | 31.54 | 2.76 | 0.28 | 1.81 |
| 6 | 10.00 | 51.84 | 1.58 | 0.61 | 0.37 | 29.48 | 2.54 | 0.37 | 3.21 |
| 7 | 9.99 | 52.15 | 0.91 | 0.63 | 0.40 | 30.20 | 2.63 | 0.15 | 2.94 |
| 8 | 9.50 | 52.21 | 0.65 | 0.83 | 0.59 | 29.15 | 3.65 | 0.11 | 3.31 |
| 9 | 10.03 | 52.09 | 0.48 | 0.67 | 0.37 | 31.59 | 2.77 | 0.10 | 1.90 |
| 10 | 9.77 | 51.94 | 0.58 | 0.74 | 0.44 | 30.57 | 3.05 | 0.13 | 2.78 |
| 11 | 9.58 | 51.81 | 0.48 | 0.65 | 0.34 | 32.83 | 2.60 | 0.11 | 1.60 |
| 12 | 9.74 | 51.75 | 0.57 | 0.78 | 0.47 | 30.44 | 3.32 | 0.10 | 2.83 |
| 13 | 9.45 | 51.71 | 0.51 | 0.71 | 0.44 | 31.44 | 3.11 | 0.09 | 2.54 |
| 14 | 9.54 | 52.44 | 0.61 | 0.69 | 0.40 | 31.77 | 2.64 | 0.13 | 1.78 |
| 15 | 9.72 | 52.00 | 0.32 | 0.68 | 0.32 | 33.06 | 2.39 | 0.09 | 1.42 |
| 16 | 9.55 | 52.30 | 1.27 | 0.66 | 0.37 | 30.36 | 2.62 | 0.12 | 2.75 |
| 17 | 9.76 | 52.08 | 0.45 | 0.73 | 0.46 | 29.78 | 3.11 | 0.11 | 3.52 |
| 18 | 9.66 | 51.92 | 0.50 | 0.81 | 0.52 | 30.12 | 3.55 | 0.08 | 2.84 |
| 19 | 9.88 | 52.44 | 0.40 | 0.67 | 0.38 | 31.54 | 2.38 | 0.10 | 2.21 |
| 20 | 9.72 | 52.20 | 0.61 | 0.73 | 0.46 | 29.70 | 2.96 | 0.14 | 3.48 |
| Min. | 9.32 | 51.71 | 0.32 | 0.61 | 0.32 | 29.15 | 2.38 | 0.08 | 1.42 |
| Max. | 10.00 | 52.87 | 1.58 | 0.83 | 0.59 | 33.06 | 3.65 | 0.51 | 3.52 |
| Average | 9.66 | 52.09 | 0.65 | 0.71 | 0.42 | 30.80 | 2.90 | 0.15 | 2.56 |

**Table S10.** Results of microprobe measurements of Ce, Nd, Sm, Dy, Gd, Y and Sc in the second micro-area of the LD11 (ppm).

| Point No. | Y | Sc | Dy | Ce | Nd | Sm | Gd |
|---|---|---|---|---|---|---|---|
| 1 | 3 | - | - | - | 16 | - | - |
| 2 | - | - | - | 47 | - | 1 | 13 |
| 3 | 6 | - | - | 18 | - | - | 15 |
| 4 | - | 3 | 129 | 5 | - | - | - |
| 5 | - | - | - | - | 14 | 3 | - |
| 6 | - | - | - | 16 | 12 | 12 | 21 |
| 7 | - | - | - | - | 22 | - | - |
| 8 | - | - | - | 59 | - | - | 8 |
| 9 | 3 | - | - | 23 | 11 | - | 20 |
| 10 | 13 | 4 | 103 | 27 | - | 19 | 19 |
| 11 | - | - | - | - | 18 | - | 44 |
| 12 | - | - | - | 33 | - | 19 | - |
| 13 | - | - | 70 | 26 | 29 | 15 | - |
| 14 | - | - | - | - | - | - | - |
| 15 | - | - | - | - | 1 | - | - |
| 16 | - | - | - | 30 | - | - | - |
| 17 | - | - | - | 28 | - | - | - |
| 18 | - | 1 | 122 | 10 | 9 | - | - |
| 19 | - | - | - | 12 | - | - | 10 |
| 20 | - | - | - | 12 | - | 11 | - |
| Min. | - | - | - | - | - | - | - |
| Max. | 13 | 4 | 129 | 59 | 29 | 32 | 44 |
